# The effects of an app to prevent negative outcomes of cyberbullying: A cluster randomized controlled trial

**DOI:** 10.1371/journal.pdig.0000819

**Published:** 2025-04-22

**Authors:** Helene Høgsdal, Sabine Kaiser, Geraldine Mabille, Kyrre Breivik, Frode Adolfsen, Monica Martinussen, Henriette Kyrrestad

**Affiliations:** 1 Regional Centre for Child and Youth Mental Health and Child Welfare - North, UiT The Arctic University of Norway, Tromsø, Norway; 2 Regional Centre for Child and Youth Mental Health and Child Welfare - West, Norwegian Research Center AS, Bergen, Norway; The University of Hong Kong, HONG KONG

## Abstract

Experiencing cyberbullying and negative incidents online can negatively affect adolescents’ mental health and well-being. NettOpp is a mobile application aiming to reduce the harmful effects of cyberbullying and negative incidents online on mental health outcomes. To evaluate the effect of the mobile app, a cluster randomized controlled trial design with three measurement points was conducted. Thirty-two Norwegian primary and secondary schools were randomly assigned to either an intervention group where the pupils (*n* = 259) got access to NettOpp during the study period, or to a waiting-list control group where the pupils (*n* = 327) got access to NettOpp after the study period. No significant findings were found in the intention-to-treat analyses, but the results from per-protocol analyses showed a decrease in hyperactivity problems among the adolescents that had used the app (*F*(4,1585) = 2.89, *p* =.021). Users of the app reported being more exposed to negative incidents online during the study period (*F*(2,1591) = 3.94, *p* =.020)*.* The frequency of cyberbullying decreased during the study period, but among all study participants, including those who had not used the app. The findings provide valuable insight into whether mobile apps can function as preventive and supportive self-help resources in reducing the harmful effects of cyberbullying and negative online incidents on mental health outcomes among adolescents.

## Author summary

Cyberbullying and other negative incidents online can have negative consequences for adolescents’ mental health and well-being. There is a need for easily accessible tools that adolescents can use to reduce the harmful effects of being exposed to negative events online. In this study, we investigated the effect of the app NettOpp on mental health outcomes, often associated with or affected by cyberbullying, for example well-being, trauma, and hyperactivity. In addition, we investigated whether NettOpp had the potential to reduce cyberbullying and other negative incidents online. While no significant effects were found in the intention-to-treat analyses, the results from per-protocol analyses revealed that hyperactivity decreased among the adolescents who had used the app during the study period. At the same time, we found that the adolescents who used the app, reported experiencing more negative incidents online compared to the adolescents that did not use the app. The frequency of negative online incidents and cyberbullying decreased among all study participants during the study period. The results of this study can provide important information regarding the preventive effect of a mobile app aimed to reduce the harmful effects of cyberbullying. The results may be particularly useful for adolescents who want to use digital tools for their mental health, as well as for app developers who want to develop apps aimed at reducing the negative consequences of being exposed to cyberbullying and negative incidents online among adolescents.

## Introduction

Cyberbullying can be defined as *“An aggressive, intentional act carried out by a group or individual, using electronic forms of contact, repeatedly and over time against a victim who cannot easily defend him or herself”* [1, p. 376]*.* Many adolescents encounter this type of bullying [2], due to their frequent use of electronic devices that enable social communication and interactions*.* The actions that young people can engage in or experience includes both active actions such as online harassment [1,3,4], or passive actions, such as exclusion and being ignored by others [4–6]. Exposure to cyberbullying and negative incidents online can have adverse consequences for adolescents’ well-being and mental health [7–9]. It is therefore important to develop useful interventions aimed at adolescents to reduce the harmful effects of cyberbullying and negative online events.

### Cyberbullying among Adolescents

The frequency rates of cyberbullying victimization vary across different study samples [e.g., 10–12]. This might be due to real differences in rates between countries, but also due to how cyberbullying is defined and measured [2,13]. In Norway, an annual school survey measures how often adolescents have been bullied in the last few months. The latest report indicates that approximately 4.5% of the children in 5^th^ grade (pupils aged 10 to 11 years) and 2.7% of adolescents in secondary schools report that they have experienced cyberbullying more than once a month [14]. Furthermore, in recent years there has been an increasing trend in the incidence of cyberbullying among adolescents [15], including in Norway [14,16]. In addition to cyberbullying, even more adolescents are experiencing negative incidents online. These are harmful online actions that are not defined as cyberbullying, often because they do not occur repeatedly over time. Estimates in Norway indicate that 33% of adolescents between the ages of 10-12 years have experienced at least one negative incident online during the last months [17].

Being a victim of cyberbullying and negative incidents online can lead to several negative consequences. For example, cyberbullying victimization is positively related to students’ academic difficulties, and it is common for cyberbullying victims to feel unsafe and lack a sense of belonging in the school environment [8,18,19]. Furthermore, being a victim of cyberbullying also contributes to an increased chance of developing mental health disorders and ailments such as anxiety, depression, sleep disturbances, psychological distress, and an overall reduced quality of life [7,9,20,21]. Cyberbullying is also linked to both suicidal ideation and attempts [22–24] and psychosomatic health difficulties such as stomachaches, back pain, dizziness and headaches [9].

### Cyberbullying Interventions

Several anti-cyberbullying interventions have shown promising effects in reducing cyberbullying victimization and perpetration [25,26]. Some interventions have also demonstrated a promising impact on increasing awareness of the potential negative consequences of cyberbullying [27]. This is important, as cyberbullying interventions should also aim to buffer the harmful effects of being cyberbullied by providing adolescents emotion-focused strategies, such as coping skills and encouragement to seek help [28]. Currently, evidence-based interventions to prevent cyberbullying are limited in Norway [29]. The Norwegian anti-bullying intervention, the Olweus-program has been found to decrease cyberbullying incidents among adolescents in the United States [30]. However, there is limited knowledge about whether these results can be generalized to Norwegian adolescents.

Most anti-cyberbullying interventions are school based [25], which means that, in many cases, an effort is required from teachers and school personnel to implement the intervention. This can be challenging to accomplish, within an already strict and limited schedule. In some studies, it has been shown that this can affect the feasibility and the effectiveness of some interventions [31,32]. Furthermore, since adolescents may have difficulties telling others that they are experiencing cyberbullying [33–35], school personnel may be unaware of which of their students are affected and in need for support [34]. Therefore, easily accessible interventions that do not require large resources from schools and among teachers are needed.

### Using mobile apps to deliver cyberbullying interventions to adolescents

Mobile apps have previously been used as platforms with the intention of improving adolescents’ mental health. The fact that they are easily accessible, cost-effective and young people’s positive attitudes towards such tools are highlighted as advantages of using them [36,37]. Cyberbullying interventions have been provided through various digital platforms, such as videos, games, websites and mobile apps [27]. A meta-analysis found that digital approaches have a small but positive impact on reducing incidents of cyberbullying [38]. It has also been shown that digital interventions have the potential to increase knowledge and awareness towards cyberbullying [27]. Furthermore, another study found that the app IMPACT increased well-being among adolescents who had experienced cyberbullying [39]. Hence, mobile apps seem to be a promising platform that can give adolescents access to cyberbullying interventions in a simple and easily accessible way. However, there is still uncertainty related to whether mobile apps can function as effective preventive and health-promoting tools for adolescents [36,40]. It is therefore important that the mobile apps are evaluated before they are offered to adolescents.

### NettOpp – a mobile application aimed to reduce the harmful effects of cyberbullying

NettOpp is a module-based app developed to support adolescents aged 11 to 16 [41]. Unlike other interventions whose main aim is to reduce cyberbullying, the primary aim of NettOpp is to reduce the harmful effects of cyberbullying and negative incidents online on mental health outcomes among adolescents. Thus, the app has a broad focus on how to cope with such negative experiences. The app consists of two modules: one psychoeducational module, and one resource module. In the psychoeducational module, the adolescents receive information about cyberbullying, emotions, possible consequences of experiencing cyberbullying, advice on what to do when experiencing cyberbullying, as well as information on Norwegian laws and regulations that apply online. The resource module consists of self-help exercises such as breathing, relaxation, and sleep exercises. This module also includes a thought-clearing exercise related to negative events online, based on cognitive behavioral therapy (CBT) and a self-confidence boost through positive push messages.

### The present study

The increasing rates of cyberbullying and the severe consequences it can have on adolescents’ mental health highlight a need for effective and easily accessible interventions. NettOpp can be an easily accessible tool for adolescents to cope with negative consequences of cyberbullying. The aim of the present study is to evaluate the effectiveness of NettOpp on its primary aims among a sample of adolescents. We hypothesized that there would be a significant difference in mental health scores during the study period between the adolescents with access to NettOpp and those randomized to a waiting list control group [41]. Additionally, the present study examines whether the use of the app can reduce cyberbullying and negative incidents online among adolescents.

## Methods

### Study design

The study was conducted with a between-subjects experimental design (intervention condition versus waiting-list control condition) with three repeated measurements (ClinicalTrials ID: NCT04176666). Data was collected through an online questionnaire at baseline (T1), two weeks after the intervention (T2), and four to six weeks after the intervention (T3).

### Recruitment and procedure

**Fig 1:**
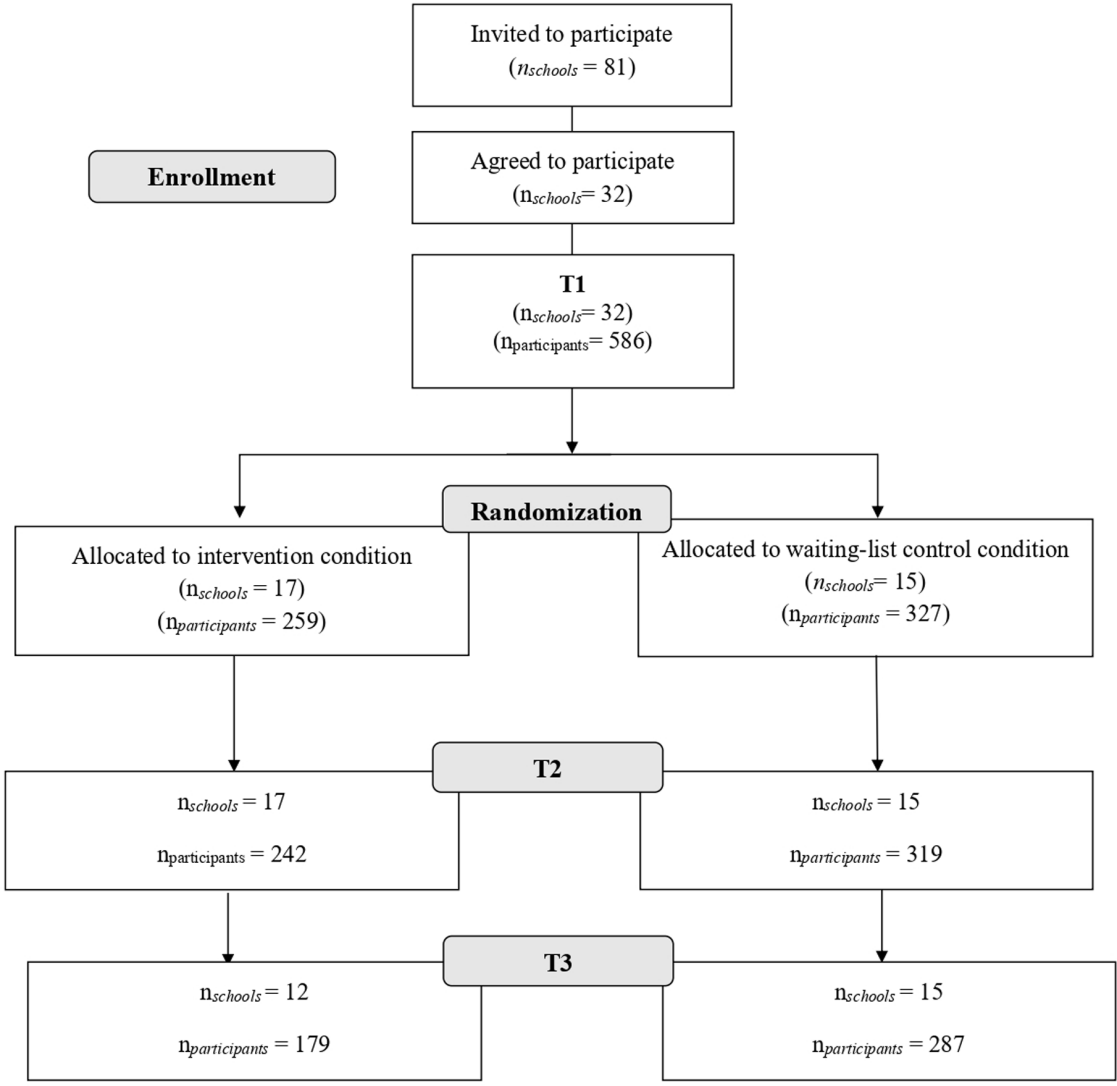
Enrollment and randomization.

Recruitment was conducted in primary and secondary schools from all regions of Norway during the spring of 2022. In Norway, primary and secondary education is free of charge, and in most cases the schools are administered by the municipalities [See also 42 for more information about the educational system in Norway]. Principals at 81 schools in Norway were contacted by phone or e-mail and were invited to participate in the study. A total of 32 schools agreed to participate (39.5% participation rate among schools; see Fig 1 for participants flow). Of those 32 schools, only two were private schools, the others were administrated by municipalities. All participating schools were responsible for forwarding information about the study to the pupils and their parents. The information included a link to an online parental consent form that all guardians with parental rights needed to sign for their adolescent to participate.

Schools that agreed to participate had to facilitate the data collection at three time points during school hours. The participants whose parents had consented to their participation were forwarded a link to an online questionnaire in Nettskjema [43]. The participants answered the questionnaire during school hours. The adolescents that did not receive parental consent worked on school-related topics on their electronic devices while the data collection took place.

### Randomization

A statistician performed a cluster randomization, where schools were assigned into the intervention group or the control group after T1. The intervention group received access to NettOpp after T1, while the control group received access to the app after T3 was completed.

### Measures

#### Socio-demographic characteristics.

Demographic information included age (in years) and sex (male, female).

#### Mental health.

Mental health was assessed using the Norwegian version of the Strength and Difficulties Questionnaire self-report (SDQ-S), the WHO-Five Well-being Index (WHO-5), and the Child and Adolescent Trauma Screening (CATS).

The SDQ-S [44,45] is a widely used instrument for assessing mental health problems among adolescents from 11 to 17 years. The SDQ consists of 25 items, distributed equally among five subscales: 1) Emotional problems scale (e.g., “I worry a lot”), 2) Conduct problems scale (e.g., “I get very angry and often lose my temper”), 3) Hyperactivity/inattention (e.g., “I am easily distracted, I find it difficult to concentrate”), 4) Peer problems (e.g., “I get along better with adults than with people my own age”), and 5) Prosocial behaviors (e.g., “I try to be nice to other people. I care about their feelings”). All responses are rated on a 3-point Likert scale from 0 to 2 (0 = not true, 1 = somewhat true, and 2 = certainly true). The subscales Emotional problems, Conduct problems, Hyperactivity/Inattention problems, and Peer problems contribute together to the Total problem scale (SDQ Total). The questionnaire is available in Norwegian and has been found to have good construct validity but mixed results when it comes to internal consistency for the different subscales [46]. In the current study only the problem scales were used and the Cronbach’s alphas were as follows for the Total problem scale: T1 α =.82, T2 α =.83, T3 α =.84; Emotional problems: T1 α =.74; T2 α =.77; T3 α =.80, Conduct Problems: T1 α =.53; T2 α =.50; T3 α =.57, Hyperactivity problems: T1 α =.72; T2 α =.74; T3 α =.76, and Peer Relationship Problems: T1 α =.58; T2 α =.58; T3 α =.59.

The WHO-5 is used to measure self-reported subjective well-being [47]. The questionnaire consists of five positively phrased items (e.g., “I have felt calm and relaxed; I woke up feeling fresh and rested”). Responses are rated on a 6-point Likert scale from 0 to 5 (0 = at no time, 1 = some of the time, 2 = less than half of the time, 3 = more than half of the time, 4 = most of the time, 5 = all of the time). The raw data, which ranges from 0 to 25 was multiplied by 4 for the sum to range from 0 (the worst imaginable well-being) to 100 (best imaginable well-being). In the current sample Cronbach’s alpha was as follows: T1 α =.83; T2 α =.82; T3 α =.88.

The CATS is a self-report screening instrument for children and adolescents from 7-17 years, which measures posttraumatic stress symptoms [48]. The instrument has been reported to have satisfactory psychometric properties [48]. The questionnaire consists of 20 statements asking how often the presented examples have bothered the adolescents in the last two weeks (e.g., “Upsetting thoughts or pictures about what happened that pop into your head”). Responses are rated on a 4-point Likert scale (0 = never, 1 = sometimes, 2 = often, 3 = almost always). Only adolescents who reported to have experienced cyberbullying in the last few months were asked to answer this instrument and were instructed to rate statements about their cyberbullying experience. Cronbach’s alpha was as follows: T1 α =.95; T2 α =.94; T3 α =.96.

#### Cyberbullying and negative incidents online.

First, the following definition of bullying was presented to the participants: Bullying is *“a) aggressive behavior or intentional “harmdoing”, b) which is carried out repeatedly and over time, c) in an interpersonal relationship characterized by an imbalance of power”* [49, p. 1173]. In addition, to make it easier for the participants to interpret the definition, examples of specific bullying behaviors were presented (e.g., ignoring or excluding someone on purpose). The participants were then asked: “How often have you been bullied *online* during the last months?”. Initially, responses were rated on a 5-point scale from 1 to 5 (1 = I have not been bullied online during the last months, 2 = only a rare time, 3 = two or three times a month, 4 = about once a week and 5 = many times a week), but were later recoded to (0 = never, 1 = 1 time or more) because only a few participants reported having been bullied more than once during the last months.

Further, the participants were asked “How often have you personally taken part in bullying one or more people online in the last few months?”. Initially, responses were rated on a 5-point scale from 1 to 5 (1 = I have not bullied anyone on the internet or mobile phone in recent months, 2 = only rarely, 3 = two or three times a month, 4 = about once a week, 5 = many times a week), but were later recoded to (0 = never, 1 = 1 time or more) because only a few adolescents answered that they had been taking part in bullying someone more than once during the last months.

Negative incidents online were measured by asking the participants how often they had experienced 13 specified individual incidents online in the last four weeks. The list of the various incidents has been used in previous studies, both in international and Norwegian contexts [35,50,51], and includes incidents such as “been ignored or excluded by others; been threatened; received a nude photo; received frightening or violent images”. Responses were rated on a 4-point scale ranging from 1 to 4 (1 = never, 2 = 1-3 times, 3 = weekly, 4 = daily) but were later recoded to (0 = never, 1 = 1 time or more) before we calculated a sum of those 13 binary variables which could range between 0 and 13.

#### Use of NettOpp.

At the second and third measurement points (T2 and T3), the participants in the intervention group were asked “how many times have you used NettOpp, in the last two weeks?” The participants were asked to rate their answer on a 7-point scale (0 times, once, 2 times, 3-5 times, 6-9 times, 10-13 times, more than 13 times).

### Ethical considerations

The study was approved by the Regional Committee for Medical and Health Research Ethics (REK North, reference 161212) and the Norwegian Agency for Shared Services in Education and Research (SIKT, reference 545417). In order to participate, the ethical committee required consent from both guardians, except in cases where there was only one guardian with parental rights for the adolescent. Participants were informed that participation in the study was voluntary and that they could at any time, without having to give any reason, choose not to answer or skip questions. The participants were also advised to contact the school nurse, a social worker, or the Red Cross Hotline in Norway if they needed someone to talk with after completing the questionnaires. School classes with a participation rate of more than 20% were compensated with a pizza lunch for the whole class.

### Statistical analyses

An a priori power analysis was conducted with PASS 16 to estimate the required sample size. The power analysis revealed that a total sample of 400 participants (200 participants in each group or 20 schools with 20 students per group) would be sufficient to detect a small to moderate effect [41].

The statistical analyses were carried out with the Statistical Package for Social Science (SPSS 27). The primary outcomes were mental health (i.e., the emotional, conduct, hyperactivity/inattention, peer problems score, and Total problem score of the SDQ-S; well-being [WHO-5 scores]; posttraumatic stress related to cyberbullying [CATS]). In addition, we examined whether the incidences of cyberbullying [binary variables] and negative online incidents [sum of 13 types of incidents] differed between groups. We used the GENLIMIXED command to run generalized linear mixed models using normal distributions for all variables, except for the binary variables estimating whether adolescents had been cyberbullied or bullied others online, which were analyzed using binomial distributions. Since our dataset included repeated measures (level 1) on the same individuals (level 2) and since individuals were further nested within schools (level 3), we included school and individuals as random intercepts in all models. In addition, we specified that the errors of the repeated measurements at the individual level followed a first-order autoregressive covariance structure, which allows the correlations between measurements of the outcome variable closer in time to be higher than the correlations between measurements further apart in time [52 p. 246-247]. An exception was the model predicting CATS where we ran a two-level model including only a random intercept for individuals because few participants answered this instrument (*n* = 185), and the model did not manage to converge with a three-level random effect structure.

In accordance with the planned analysis strategy [41], we used an *intention-to-treat approach*. That is, all participants were analyzed according to their randomized group assignment (i.e., intervention versus control group). The results of these analyses are presented in the supplementary material (See S1 Table and S2 Table for Fixed Effects Models, and S3 Table and S4 Table for the Mixed Effect Models). However, as 58.1% of the participants assigned to the intervention group had not used the app during the study period, we also conducted the analyses using a *per-protocol approach* (i.e., where the condition of the participants was coded as 0 = intervention group and reporting using the app at least once, 1 = intervention group and did not report using the app, and 2 = control group). This approach allows for a closer look at the app’s *actual* effect among the participants who used it, and it is frequently used in research on the effect of mental health apps because many respondents often do not use the intervention during the study period [53,54]. The models’ predictors were age, sex, time (categorical variable T1, T2, T3), per-protocol group, and an interaction between per-protocol group and time. Since we had no missing data for any of those predictors (i.e., age, sex, time, or condition), all answers delivered at the different time points could be included and used for the estimation of the multilevel models.

Descriptive statistics at baseline and differences between groups were analyzed using a univariate general linear model. Furthermore, we tested for a possible selective dropout using a binary regression where participation at T3 was coded as 0 = did not participate versus 1 = did participate at T3. We tested whether sex, age, and the different mental health and cyber-bullying variables measured at T1 predicted participation at T3. In all tests, a *p*-value of less than.05 was considered statistically significant.

## Results

### Descriptives

The baseline sample consisted of *N* = 586 adolescents, with 297 males (50.7%) and 289 females (49.3%), and an age range from 11 to 16 years (*M*_age_ = 12.20, *SD* = 0.94). A total of *n* = 108 (18.4%) had experienced cyberbullying, *n* = 382 (65.2%) had experienced at least one negative incident online, and *n* = 30 (6.1%) had cyberbullied others. The T2 sample included 561 adolescents, and the T3 sample included 466 adolescents. A total of 394 participants responded at all three measurement points (*n* = 150 in the intervention group and *n* = 244 in the control group).

A total of 259 respondents (49.0% males) were randomized to the intervention group and received access to NettOpp. Of those, 108 (41.7% males) *used* the app during the study period, while 151 (54.3% males) *did not* use the app. The control group consisted of 327 respondents (52.0% males). There was no statistically significant difference between the groups in experiencing cyberbullying, negative online incidents, cyberbullying others, or any of the mental health outcomes at baseline. Only age was significantly different between the two groups. That is, between the control group and the group in the intervention group that had not used the app during the study period. Descriptive statistics and differences between groups at baseline are presented in Table 1.

**Table 1:** Descriptive Statistics at Baseline.

	Intervention group						Control group			Total		
	Used app			Not used app								
	*n*	*M*	*SD*	*n*	*M*	*SD*	*n*	*M*	*SD*	*N*	*M*	*SD*
Age	108	12.3	1.0	151	**12.4** ^*^	1.2	327	**12.1** ^*^	0.7	586	12.2	0.9
SDQ Total	108	11.2	6.0	149	10.8	6.2	322	10.3	5.7	579	10.6	5.9
Emotion problems	108	3.1	2.4	149	3.0	2.6	322	2.9	2.3	579	3.0	2.4
Conduct problems	108	1.9	1.8	150	1.7	1.7	322	1.6	1.5	580	1.7	1.6
Hyper problems	108	4.4	2.2	150	4.4	2.3	322	4.0	2.3	580	4.2	2.3
Peer problems	108	1.9	1.7	149	1.8	1.5	322	1.8	1.8	579	1.8	1.7
WHO-5	108	64.1	20.8	150	63.3	20.0	324	66.1	19.3	582	65.0	19.8
CATS	20	22.4	13.1	20	18.3	15.4	51	16.6	13.4	91	18.2	13.8

***Note.*** *n* = part of the sample; *M* = mean; *SD* = standard deviation; *N* = total sample*.* Significant differences between the groups are presented in bold.

* *p* =.003

The attrition analyses identified age as the only predictor of participation at T3. The odds for participation at T3 decreased by 17% for each 1-year increase in age of the respondent. The other independent variables did not predict participation at T3 (all *p* >.155; Table 2).

**Table 2:** Attrition analyses to predict participation at T3.

Independent variables	*B*	*S.E.*	*Wald*	*df*	*p*	*Exp(B)*
Age	-0.19	0.09	4.37	1.00	**.036**	0.83
Sex	0.25	0.18	2.02	1.00	.155	1.28
SDQ Total	0.00	0.02	0.00	1.00	.989	1.00
Emotional problems	0.02	0.04	0.21	1.00	.647	1.02
Conduct problems	0.00	0.06	0.01	1.00	.939	1.00
Hyperact. problems	-0.03	0.04	0.72	1.00	.398	0.97
Peer problems	0.02	0.06	0.16	1.00	.687	1.02
WHO-5	0.00	0.01	0.57	1.00	.450	1.00
CATS	0.01	0.02	0.11	1.00	.743	1.01
Cyberbullied	-0.08	0.14	0.33	1.00	.568	0.92
Cyberbullied others	0.14	0.29	0.22	1.00	.640	1.15
Negative online incidents	0.01	0.04	0.06	1.00	.808	1.01

### Mental health

**Fig 2:**
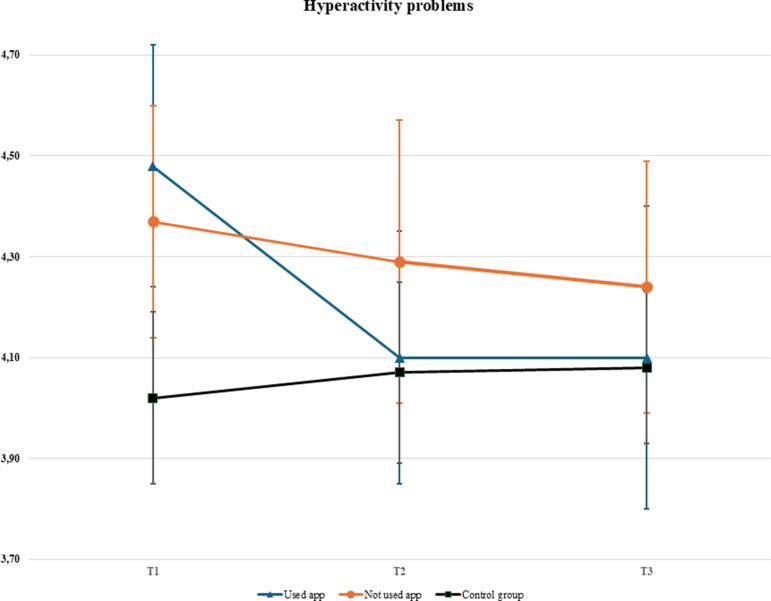
Hyperactivity problem-scores among per-protocol groups during the study period. Note. The estimates shown are calculated with the average age of the sample (M = 12.20 years).

No significant main effects between the intervention and the control group were found in the intention-to-treat analyses (see S1–S4 Tables in the supplementary files). In the per-protocol analyses, the only model where the interaction between condition and time was significant was the model predicting hyperactivity (*F*(4,1585) = 2.89, *p* =.021). Hyperactivity decreased faster over time for participants who had used the app (see Table 3 and Fig 2). All other interactions between time and condition had no significant effect on mental health outcomes (all *p* >.188). The hyperactivity problems score was also predicted by age of the respondent (*p* =.029). Sex predicted three of the SDQ subscales scores (i.e., the Total problem scale, Emotion problem scale, and Conduct problem scale), and for the WHO-5 score. That is, females had higher scores on the total problem scale (*β* = 1.45, *p* =.004), on the emotion problem scale (*β* = 1.79, *p* <.001), and scored lower than males on well-being (*β* = -9.19, *p* <.001), and on the conduct problem scale (*β* = -0.21, *p* =.033; all fixed coefficients are presented in Table 3 and Table 4).

**Table 3:** Mixed Effect Models Predicting Mental Health with the Different SDQ Subscales.

	SDQ Total			Emotion problems			Conduct problems			Hyper problems			Peer problems		
	*B*	95% CI	*p*	*B*	95% CI	*p*	*B*	95% CI	*p*	*B*	95% CI	*p*	*B*	95% CI	*p*
Age	0.39	[-0.16, 0.94]	.168	0.08	[-0.18, 0.33]	.559	0.02	[-0.13, 0.16]	.812	0.24	[0.03, 0.46]	**.029**	0.00	[-0.13, 0.13]	.981
Sex^a^	1.45	[0.45, 2.45]	**.004**	1.79	[1.41, 2.17]	**<.001**	-0.21	[-0.40, -0.02]	**.033**	-0.05	[-0.48, 0.37]	.802	-0.04	[-0.30, 0.22]	.762
Time 2^b^	-0.65	[-1.35, 0.05]	.070	-0.15	[-0.51, 0.22]	.425	0.01	[-0.27, 0.28]	.972	-0.38	[-0.67, -0.10]	**.008**	-0.10	[-0.32, 0.11]	.352
Time 3 ^b^	-0.78	[-1.95, 0.40]	.196	-0.01	[-0.44, 0.42]	.958	-0.15	[-0.50, 0.20]	.411	-0.41	[-0.79, -0.04]	**.030**	-0.24	[-0.62, 0.14]	.222
Per protocol 1^c^	-0.52	[-1.71, 0.67]	.391	0.11	[-0.38, 0.60]	. 654	-0.33	[-0.79, 0.14]	.166	-0.11	[-0.38, 0.16]	.416	-0.18	[-0.60, 0.24]	.392
Per protocol 2 ^c^	-0.67	[-2.36, 1.02]	.436	0.00	[-0.55, 0.55]	.999	-0.25	[-0.66, 0.16]	.228	-0.46	[-1.05, 0.12]	.121	-0.04	[-0.51, 0.44]	.882
Time 2* per protocol 1^d^	0.62	[-0.24, 1.48]	.156	0.10	[-0.27, 0.47]	.590	0.09	[-0.30, 0.48]	.649	0.30	[0.03, 0.57]	**.030**	0.10	[-0.24, 0.43]	.571
Time 2* per protocol 2^d^	0.49	[-0.26, 1.25]	.201	0.01	[-0.38, 0.39]	.981	0.05	[-0.24, 0.35]	.723	0.44	[0.11, 0.77]	**.009**	-0.03	[-0.31, 0.24]	.819
Time 3* per protocol 1^d^	0.34	[-0.73, 1.42]	.531	-0.27	[-0.67, 0.12]	.177	0.26	[-0.13, 0.65]	.187	0.29	[-0.03, 0.61]	.077	0.11	[-0.30, 0.53]	.591
Time 3* per protocol 2^d^	0.58	[-0.65, 1.80]	.354	-0.19	[-0.65, 0.27]	.416	0.11	[-0.26, 0.48]	.575	0.48	[0.07, 0.89]	**.022**	0.20	[-0.20, 0.60]	.326

*Note*. ^a^Males is the reference group; ^b^ Time 1 (T1) is the reference group; ^c^ Per protocol 0 (intervention group and used app) is the reference group; ^d^ all the others are reductant.

**Table 4:** Mixed Effect Models Predicting Mental health. Cyberbullying, Bullying Others and Negative Online Incidents.

	WHO-5			CATS				Cyberbullied			Cyberbullied others			Negative online incidents	
	*B*	95% CI	*p*	*B*	95% CI	*p*	*B*	95% CI	*p*	*B*	95% CI	*p*	*B*	95% CI	*p*
Age	-1.26	[-2.68, 0.16]	.082	1.67	[-0.98, 4.32]	.216	-0.05	[-0.38, 0.28]	.754	0.13	[-0.20, 0.47]	.431	0.06	[-0.16, 0.28]	.591
Sex^a^	-9.19	[-12.83, -5.56]	**<.001**	3.20	[-1.35, 7.74]	.167	0.18	[-0.33, 0.69]	.490	-0.99	[-1.92, -0.06]	**.037**	-0.02	[-0.41, 0.37]	.912
Time 2^b^	0.06	[-3.32, 3.45]	.970	-0.80	[-7.66, 6.07]	.819	-0.92	[-1.80, -0.05]	.**039**	-0.35	[-3.41, 2.71]	.825	-0.10	[-0.52, 0.32]	.635
Time 3 ^b^	1.13	[-2.93,5.19]	.585	-3.85	[-9.56, 1.87]	.185	-1.47	[-2.76, -0.17]	**.026**	-1.22	[-3.88, 1.45]	.370	-0.35	[-0.83, 0.13]	.151
Per protocol 1^c^	-0.88	[-4.87, 3.10]	.664	-2.43	[-10.25, 5.38]	.540	-0.69	[-1.48, 0.10]	.088	-0.60	[-2.32, 1.12]	.496	-0.57	[-1.12, -0.02]	**.042**
Per protocol 2 ^c^	1.16	[-3.85, 6.18]	.649	-4.09	[-9.96, 1.78]	.171	-0.68	[-1.69, 0.34]	.191	-0.12	[-2.15, 1.90]	.904	-0.16	[-1.02, 0.70]	.712
Time 2* per protocol 1^d^	0.91	[-1.77, 3.58]	.505	4.08	[-11.11,19.27]	.597	-0.87	[-2.56, 0.82]	.313	0.73	[-2.09, 3.54]	.613	-0.15	[-0.52, 0.23]	.444
Time 2* per protocol 2^d^	1.37	[-2.52, 5.27]	.489	5.13	[-2.97, 13.22]	.213	-0.13	[-1.54, 1.28]	.855	-0.60	[-3.91, 2.71]	.722	-0.07	[-0.53, 0.39]	.760
Time 3* per protocol 1^d^	-2.37	[-5.88, 1.14]	.186	1.97	[-9.50, 13.44]	.735	-0.26	[-2.48, 1.97]	.820	-0.42	[-3.02, 2.18]	.751	-0.01	[-0.36, 0.35]	.957
Time 3* per protocol 2^d^	0.73	[-3.49, 4.95]	.735	2.25	[-6.06, 10.56]	.594	0.20	[-1.30, 1.70]	.792	0.48	[-2.40, 3.37]	.743	-0.04	[-0.57, 0.50]	.894

*Note*. WHO-5 = World Health Organization-Five Well-being Index; CATS = Child and Adolescent Trauma Screening; ^a^Males is the reference group; ^b^ Time 1 (T1) is the reference group; ^c^ Per protocol 0 (intervention group and used app) is the reference group; ^d^ all the others are reductant.

### Cyberbullying and negative online incidents

There was no significant interaction effect between time and condition in the models predicting cyberbullying and negative online incidents (all *p* >.158). However, we found a significant relationship between the per protocol group and the amount of experienced negative online incidents (*F*(2,1591) = 3.94, *p* =.020)*.* More specifically, the participants that were in the intervention group, but did not use the app reported fewer negative online incidents compared to the participants in the intervention group that used the app (*β= -0.57*, *p* =.042, see Table 4). In addition, frequency of negative online incidents (*F*(2,1591) = 3.67, *p* =.026), cyberbullying (*F*(2,1602) = 17.25, *p* <.001) and the odds of bullying others online (*F*(2,1587) = 3.60, *p* =.028) decreased over time for all the participants. Sex was related to having bullied others online (*F*(1,1587) = 4.36, *p* =.037), with males being more likely to bully others online (*β* = -0.99, *p* =.037; see Table 4).

## Discussion

The aim of the current study was to evaluate the effect of NettOpp on mental health outcomes commonly associated with cyberbullying and negative online incidents. Furthermore, NettOpp’s potential to reduce the incidence of cyberbullying among adolescents was investigated.

There was a significant reduction in hyperactivity among users of NettOpp. The observed reduction is a positive and promising finding, as both cyberbully victims and adolescents who cyberbully others have shown higher scores on hyperactivity and inattention [20,55]. The majority of the research on the consequences of high hyperactivity scores among adolescents has been conducted on adolescents with an attention deficit hyperactivity disorder [ADHD; 56]. However, high levels of hyperactivity can have negative consequences,even for adolescents who do not meet the full diagnostic criteria for ADHD [57]. That is, hyperactivity problems are linked to poorer school performance and lower school completion rate [58,59] and feeling unsafe at school [19]. Young people with higher scores of hyperactivity in childhood also have a higher risk of experiencing developmental problems in adolescence and developing psychiatric diagnoses in adulthood [60]. It is difficult to interpret why NettOpp had an effect on the users’ hyperactivity but not on the other mental health outcomes. It is possible that the information about emotions and emotion regulation may have helped the adolescents develop skills to better cope with hyperactivity. Moreover, both mindfulness and CBT have been shown to reduce hyperactivity in young people [61,62]. It is therefore conceivable that the meditation exercises and the thought-clearing exercise in particular, may have had an effect on the adolescents’ hyperactivity. Especially the meditation exercises in the app have a strong focus on relaxation and give the adolescents the opportunity to do specific breathing exercises, listen to relaxing music, and think about something they are grateful for. It has also been shown people with high hyperactivity scores often find technological solutions satisfying [63,64], which suggests that NettOpp could be a promising tool for reducing hyperactivity in adolescents. Furthermore, while no other significant findings were observed in the remaining mental health outcomes examined in this study, it is important to consider that NettOpp was evaluated in a general adolescent population and in a regular school setting. This sample included pupils who had experienced cyberbullying and negative online incidents, as well as those who had not. Having found an effect on the hyperactivity scores in this sample is a promising result, as universal interventions that yield small effects on an individual level can be beneficial for the broader population [65,66].

The findings also indicate that the app users reported being more exposed to negative online incidents throughout the study period, compared to the two other groups. One possible explanation for this could be that the adolescents that experienced negative online incidents were more likely to use the app during the study period. Another possible explanation could be that the use of NettOpp may have increased the users’ knowledge about cyberbullying and negative incidents online, making them more likely to report such events after using the app. In a previous user evaluation of the app, the respondents anticipated that NettOpp would be able to increase adolescents’ knowledge on cyberbullying and negative online incidents [67]. The app’s psychoeducational module provides information about what negative online incidents are, and it highlights rights and legal considerations related to how to behave online. The app users learn about potential issues with common actions such as “posting pictures of friends online without their consent” or “writing mean or harmful comments intended as a joke”. Consequently, the app users may have gained a broader understanding of the various occurrences that can be classified as negative online incidents. This is important as increasing adolescents’ knowledge and awareness may contribute to reducing cyberbullying, as well as enhance their ability to cope with such incidents [27].

Another finding of the study was that both cyberbullying and negative incidents online decreased among all three groups during the study period. This could be related to an increased awareness of cyberbullying among the participants during the study period. It is a positive finding as cyberbullying is a significant contributor to poorer mental health and well-being [9].

### Strengths and limitations

A strength of this study is that the effect of a mobile app, developed with aim to reduce the harmful effects of cyberbullying and negative incidents online on mental health outcomes, was measured using a randomized controlled trial design. The findings can contribute to a broader understanding of how to develop effective mental health apps to prevent the negative consequences of cyberbullying. However, several limitations must be considered when interpreting the results.

First and foremost, the investigation of multiple hypotheses in a single study can increase the probability of a false positive finding [Type I error; 68]. In this study, the effect of NettOpp was measured on 10 outcome variables. Thus, it is possible that the significant finding of a reduction in hyperactivity among users of the app could represent a Type I error. However, the proportion of adolescents that had used the app was smaller than what the power analysis estimated would be needed to detect a small effect of the intervention [41]. Therefore, adjusting the significance level for multiple hypotheses could have further reduced the statistical power of the study. Alternatively, a significance level at 1% could have been used [69]. However, this adjustment of the significance level comes with the disadvantage that the power is reduced and thus increases the probability of a false negative [Type II error; 70]. Therefore, the analytic strategy plan for the main objectives of the app were conducted in line with what was specified in the protocol article [41].

Another limitation is that only about 40% of the adolescents randomized to the intervention group reported that they had used the app. Low user engagement is common among users of mental health apps [54,71]. Due to that reason, per-protocol analyses were conducted in addition to the intention-to-treat analyses that were initially planned. This approach might be beneficial to provide an estimation of the actual efficacy of an intervention [53,72]. However, it is also important to emphasize that by using a per-protocol approach, one loses the benefits associated with randomization, and that this approach can disclose an overstated effect of the intervention [72]. Thus, the significant finding of a reduction in hyperactivity should be interpreted carefully, both due to the posssibility of a potential Type I error, and also due to the possibility of an an overstated effect of the intervention caused by the use of per-protocol analyses.

It is also important to consider the sample in the study. First and foremost, younger participants were more likely to participate at all measurement points. The results are therefore based on a younger sample than the intended target group, which was aged 11 to 16 years. Furthermore, obtaining parental consent was challenging. In some cases, we were not able to obtain consent from both guardians (e.g., due to shift work over several weeks or a lack of technical skills). It has previously been shown that more educated parents, with higher socio-cultural status, consent more often to participation in research on behalf of their children [73–76]. It is possible that a similar bias may have occurred in this study, and that participants who might have benefited more from the intervention were excluded from the study due to the consent process. However, collecting consent from all guardians was a requirement from the ethical committee that assessed our study, and we were obliged to follow that recommendation.

Furthermore, the fact that the results are based solely on self-report might have led to potential bias. It is especially important to consider that the adolescents responded to the questionnaires during school hours. Even though the school personnel was instructed to facilitate the data collection so that the adolescents could answer individually, some might have had concerns about answering honestly in a setting where their classmates and friends were present.

The scales used in the study have been used in research examining the consequences of cyberbullying previously [e.g., the SDQ; 20]. However, a limitation of the study may be that the scales were not specifically designed to assess distress or outcomes directly caused by cyberbullying. It could have been advantageous to use other instruments that are developed to measure psychological aspects that can be triggered by cyberbullying [e.g., 77]. Furthermore, both the conduct scale and the peer problem scale from the SDQ had low internal consistency, which has also been reported in previous research conducted in Scandinavia [46]. However, the other subscales had at least acceptable reliability and a psychometric review of the SDQ in a Scandinavian context recommends the instrument to be used in research on adolescents’ mental health [46].

### Future research

Future research on the effectiveness of NettOpp on mental health outcomes and cyberbullying should include a design that ensures a sufficient participation rate, so that the app’s effect can be better examined with an intention-to-treat approach. This also includes ensuring that the participants in the intervention group use the app as intended. More frequent reminders to download the app and to use it during the study period could be beneficial to encourage more young people to use the app [54]. Furthermore, including qualitative components, like interviews, could help to further investigate how and why adolescents did or did not use the app. Particularly, it could be beneficial to clarify why some of the adolescents did not use the app, despite being given access to it, and what changes they would recommend to make it more likely that they would use the app. At the same time, it should be investigated what causes some people to choose to use these tools while others do not. This can help to develop more suitable apps for adolescents, which facilitate them to be more willing to use them.

As many more participants reported having been exposed to negative online incidents compared to being cyberbullied, future research should investigate the relation between self-reported single negative incidents online and being cyberbullied. Additionally, further research should investigate whether knowledge about this topic (i.e., what is classified as cyberbullying and negative online incidents) influences how adolescents themselves assess the negative events they experience online.

As the study did not specifically target adolescents who had experienced cyberbullying or negative online incidents, the effect of NettOpp on this group is still uncertain and should be investigated in further research. This could contribute to a greater understanding of whether NettOpp is more effective in buffering the negative consequences of cyberbullying and negative incidents online. Further evaluation of NettOpp should also examine the app’s effect from a perspective other than that of adolescents, for example from health nurses, teachers, or psychologists who also work with young people exposed to cyberbullying and negative online incidents.

## Conclusion

In this study, the effectiveness of NettOpp, a mobile application designed to support young people who experience negative incidents online, was assessed through a cluster randomized trial. While the intention to treat analyses showed no statistically significant effect, the per-protocol analyses revealed that participants who used the app showed a decrease in hyperactivity scores during the study period. This might be an important finding as high levels of hyperactivity and inattention can have negative consequences such as poorer school performance and greater chances of developing psychiatric disorders later in life. Furthermore, users of the app reported being more exposed to negative incidents online during the study period. This may suggest that the app either increases young people’s awareness of what constitutes negative incidents online or that they are more likely to use such tools when they experience negative events online. However, NettOpp did not show an effect on the other mental health outcomes, nor on the incidence of cyberbullying. The results on whether NettOpp can function as a preventive tool on these outcomes are therefore limited and should be investigated further. Future studies with larger sample sizes should be conducted to explore other benefits of the app for adolescents dealing with cyberbullying and negative online incidents.

## Supporting information

S1 Table Fixed Effect Models Predicting Mental Health with the Different SDQ Subscales. (DOCX)

S2 Table Fixed Effect Models Predicting Mental health, Cyberbullying, Cyberbullied Others and Negative Online Incidents. (DOCX)

S3 Table Mixed Effect Models Predicting Mental Health with the Different SDQ Subscales. (DOCX)

S4 Table Mixed Effect Models Predicting Mental health, Cyberbullying, Bullying Others and Negative Incidents Online. (DOCX)
