# Supplementary material for: The effects of an app to prevent negative outcomes of cyberbullying: A cluster randomized controlled trial
Source: PLOS Digit Health. 2025 Apr 22;4(4):e0000819. doi: 10.1371/journal.pdig.0000819 (PMC12013879; doi:10.1371/journal.pdig.0000819)
Supplement: S1 Table — (DOCX) [file pdig.0000819.s001.docx]

**Table S1**

*Fixed Effects Models Predicting Mental health with the Different SDQ Subscales*

|  | **SDQ total** | | | **Emotion problems** | | | **Conduct problems** | | | **Hyper problems** | | | **Peer problems** | | |
| --- | --- | --- | --- | --- | --- | --- | --- | --- | --- | --- | --- | --- | --- | --- | --- |
|  | F | *df* | *p* | F | *df* | *p* | F | *df* | *p* | F | *df* | *p* | F | *df* | *p* |
| Age | 1.87 | 1(1586) | .172 | 0.35 | 1(1589) | .555 | 0.05 | 1(1590) | .821 | 4.73 | 1(1588) | **.030** | 0.00 | 1(1587) | .979 |
| Sex | 8.47 | 1(1586) | **.004** | 85.65 | 1(1589) | **<.001** | 4.04 | 1(1590) | **.045** | 0.08 | 1(1588) | .784 | 0.06 | 1(1587) | .811 |
| Time | 1.73 | 2(1586) | .177 | 2.32 | 2(1589) | .099 | 1.60 | 2(1590) | .201 | 0.75 | 2(1588) | .474 | 1.53 | 2(1587) | .217 |
| Condition | 0.05 | 1(1586) | .818 | 0.23 | 1(1589) | .630 | 0.14 | 1(1590) | .704 | 0.47 | 1(1588) | .493 | 0.20 | 1(1587) | .655 |
| Time*Condition | 0.53 | 2(1586) | .589 | 0.08 | 2(1589) | .919 | 0.04 | 2(1590) | .965 | 2.20 | 2(1588) | .112 | 1.80 | 2(1587) | .165 |

*Note*. *P*-values that are <.05 are bolded.
