# Supplementary material for: The effects of an app to prevent negative outcomes of cyberbullying: A cluster randomized controlled trial
Source: PLOS Digit Health. 2025 Apr 22;4(4):e0000819. doi: 10.1371/journal.pdig.0000819 (PMC12013879; doi:10.1371/journal.pdig.0000819)
Supplement: S2 Table — (DOCX) [file pdig.0000819.s002.docx]

**Table S2**

*Fixed effects Models Predicting Mental health, Cyberbullying, Cyberbullying Others and Negative Incidents Online*

|  | **WHO-5** | | | **CATS** | | | **Cyberbullied** | | | **Cyberbullied others** | | | **Negative online incidents** | | |
| --- | --- | --- | --- | --- | --- | --- | --- | --- | --- | --- | --- | --- | --- | --- | --- |
|  | F | *df* | *p* | F | *df* | *p* | F | *df* | *p* | F | *df* | *p* | F | *df* | *p* |
| Age | 3.00 | 1(1595) | .083 | 1.51 | 1(177) | .221 | 0.07 | 1(1605) | .791 | 0.58 | 1(1590) | .447 | 0.29 | 1(1594) | .592 |
| Sex | 24.03 | 1(1595) | **<.001** | 1.92 | 1(177) | .167 | 0.85 | 1(1605) | .357 | 4.34 | 1(1590) | **.037** | 0.01 | 1(1594) | .925 |
| Time | 0.89 | 2(1595) | .409 | 2.33 | 2(177) | .100 | 12.53 | 2(1605) | **<.001** | 2.83 | 2(1590) | .059 | 5.01 | 2(1594) | **.007** |
| Condition | 1.67 | 1(1595) | .197 | 0.32 | 1(177) | .571 | 0.05 | 1(1605) | .823 | 0.03 | 1(1590) | .864 | 0.31 | 1(1594) | .576 |
| Time*Condition | 0.53 | 2(1595) | .587 | 0.41 | 2(177) | .663 | 0.14 | 2(1605) | .872 | 1.14 | 2(1590) | .230 | 0.04 | 2(1594) | .963 |

*Note*. *P*-values that are <.05 are bolded.
