# Supplementary material for: The effects of an app to prevent negative outcomes of cyberbullying: A cluster randomized controlled trial
Source: PLOS Digit Health. 2025 Apr 22;4(4):e0000819. doi: 10.1371/journal.pdig.0000819 (PMC12013879; doi:10.1371/journal.pdig.0000819)
Supplement: S3 Table — (DOCX) [file pdig.0000819.s003.docx]

**Table S3**

*Mixed Effect Models Predicting Mental Health with the Different SDQ Subscales*

|  | **SDQ total** | | | **Emotion problems** | | | **Conduct problems** | | | **Hyper problems** | | | **Peer problems** | | |
| --- | --- | --- | --- | --- | --- | --- | --- | --- | --- | --- | --- | --- | --- | --- | --- |
|  | *B* | 95% CI | *p* | *B* | 95% CI | *p* | *B* | 95% CI | *p* | *B* | 95% CI | *p* | *B* | 95% CI | *p* |
| Age | 0.39 | [-0.17, 0.94] | .172 | 0.08 | [-0.18, 0.33] | .547 | 0.02 | [-0.13, 0.16] | .821 | 0.24 | [0.02, 0.46] | **.030** | 0.01 | [-0.13, 0.13] | .979 |
| Sex^a^ | 1.47 | [0.48, 2.45] | **.004** | 1.78 | [1.41, 2.16] | **<.001** | -0.19 | [-0.38, -0.01] | **.045** | -0.06 | [-0.48, 0.36] | .784 | -0.03 | [-0.30, 0.24] | .811 |
| Time 2^b^ | -0.15 | [-0.46, 0.15] | .324 | -0.14 | [-0.29, 0.01] | .056 | 0.06 | [-0.06, 0.17] | .317 | 0.06 | [-0.11, 0.23] | .502 | -0.14 | [-0.30, 0.03] | .110 |
| Time 3^b^ | -0.20 | [-0.56, 0.16] | .280 | -0.20 | [-0.37, -0.03] | **.019** | -0.04 | [-0.18, 0.09] | .553 | 0.07 | [-0.11, 0.24] | .453 | -0.04 | [-0.16, 0.09] | .576 |
| Condition^c^ | 0.36 | [-1.18, 1.90] | .644 | 0.07 | [-0.39, 0.52] | .775 | 0.06 | [-0.30, 0.42] | .750 | 0.40 | [-0.15, 0.95] | .156 | -0.07 | [-0.49, 0.34] | .727 |
| Time 2*condition^d^ | -0.15 | [-0.69, 0.39] | .579 | 0.05 | [-0.23, 0.33] | .723 | 0.00 | [-0.18, 0.18] | .997 | -0.28 | [-0.61, 0.06] | .104 | 0.09 | [-0.16, 0.33] | .484 |
| Time 3*condition^d^ | -0.38 | [-1.19, 0.43] | .353 | 0.06 | [-0.25, 0.38] | .697 | 0.03 | [-0.24, 0.30] | .823 | -0.33 | [-0.64, -0.02] | **.038** | -0.14 | [-0.40, 0.12] | .293 |

*Note*. ^a^Males are the reference group; ^b^ Time 1 (T1) is the reference group; ^c^ Condition 0 (control group) is the reference group; ^d^ all the others are reductant. *P*-values that are <.05 are bolded.
