# Supplementary material for: The effects of an app to prevent negative outcomes of cyberbullying: A cluster randomized controlled trial
Source: PLOS Digit Health. 2025 Apr 22;4(4):e0000819. doi: 10.1371/journal.pdig.0000819 (PMC12013879; doi:10.1371/journal.pdig.0000819)
Supplement: S4 Table — (DOCX) [file pdig.0000819.s004.docx]

**Table S4**

*Mixed Effect Models Predicting Mental health. Cyberbullying. Bullying Others and Negative Incidents Online*

|  | **WHO-5** | | | **CATS** | | | **Cyberbullied** | | | **Cyberbullied others** | | | **Negative online incidents** | | |
| --- | --- | --- | --- | --- | --- | --- | --- | --- | --- | --- | --- | --- | --- | --- | --- |
|  | *B* | 95% CI | *p* | *B* | 95% CI | *p* | *B* | 95% CI | *p* | *B* | 95% CI | *p* | *B* | 95% CI | *p* |
| Age | -1.26 | [-2.69, 0.17] | .083 | 1.65 | [-1.00, 4.30] | .221 | -0.05 | [-0.39, 0.30] | .791 | 0.13 | [-0.20, 0.46] | .447 | 0.06 | [-0.16, 0.29] | .592 |
| Sex^a^ | -9.12 | [-12.76, -5.47] | **<.001** | 3.19 | [-1.35, 7.74] | .167 | 0.23 | [-0.27, 0.73] | .357 | -0.98 | [-1.90, -0.06] | .**037** | 0.02 | [-0.37, 0.41] | .925 |
| Time 2 ^b^ | 1.44 | [-0.44, 3.32] | .134 | 4.30 | [0.09, 8.50] | **.045** | -1.05 | [-2.15, 0.05] | .060 | -0.95 | [-2.21, 0.31] | .141 | -0.17 | [-0.37, 0.02] | .084 |
| Time 3 ^b^ | 1.86 | [0.71, 3.01] | **.010** | -1.66 | [-7.71, 4.39] | .588 | -1.26 | [-2.02, -0.51] | **.010** | -0.74 | [-1.85, 0.37] | .193 | -0.39 | [-0.63, -0.14] | **.002** |
| Condition^c^ | -1.69 | [-5.75, 2.37] | .414 | 2.91 | [-2.26, 8.08] | .268 | 0.27 | [-0.71, 1.25] | .589 | -0.22 | [-1.83, 1.39] | .791 | -0.19 | [-0.91, 0.54] | .611 |
| Time 2*condition^d^ | -0.87 | [-4.15, 2.41] | .604 | -3.50 | [-11.11, 4.12] | .366 | -0.22 | [-1.48, 1.04] | .736 | 1.02 | [-1.65, 3.70] | .453 | -0.01 | [-0.38, 0.38] | .995 |
| Time 3*condition^d^ | -1.82 | [-5.28, 1.64] | .302 | -1.28 | [-9.17, 6.61] | .749 | -0.31 | [-1.48, 0.86] | .609 | -0.63 | [-2.45, 1.19] | .500 | 0.04 | [-0.42, 0.51] | .853 |

*Note*. WHO-5 = World Health Organization-Five Well-being Index; CATS = Child and Adolescent Trauma Screening; ^a^Males are the reference group; ^b^ Time 1 (T1) is the reference group; ^c^ Condition 0 (control group) is the reference group; ^d^ all the others are reductant. *P*-values that are <.05 are bolded.
